# Sinuous Is a Claudin Required for Locust Molt in *Locusta migratoria*

**DOI:** 10.3390/genes15070850

**Published:** 2024-06-27

**Authors:** Yichao Zhang, Hongjing Li, Qiuyan Lan, Xiaoman Liu, Haihua Wu, Jianzhen Zhang, Xiaoming Zhao, Yanli Wang

**Affiliations:** 1 Institute of Applied Biology, Shanxi University, Taiyuan 030006, China; zyc1991@sxu.edu.cn (Y.Z.); lihongjing813@163.com (H.L.); l15735853863@163.com (Q.L.); xiaomanl0725@163.com (X.L.); wuhaihua04@sxu.edu.cn (H.W.); zjz@sxu.edu.cn (J.Z.); zxming@sxu.edu.cn (X.Z.); 2 Shanxi Key Laboratory of Nucleic Acid Biopesticides, Taiyuan 030006, China; 3 College of Life Science, Shanxi University, Taiyuan 030006, China

**Keywords:** *L. migratoria*, *sinuous*, molt, septate junction

## Abstract

The epidermal cells of insects are polarized epithelial cells that play a pivotal role in the insect’s molting process. Sinuous, a pivotal structural protein involved in the formation of septate junctions among epithelial cells, is essential for its physiological function. In this study, to determine whether sinuous participates in the regulation of insect molting, we identified the sinuous gene, *Lmsinu*, in *Locusta migratoria*, which encodes a protein belonging to the claudin family and shares 62.6% identity with *Drosophila*’s sinuous protein. *Lmsinu* is expressed in multiple tissues, and its expression level in the integument significantly increases prior to molting. Knockdown of *Lmsinu* in *L. migratoria* results in larval mortality during molting. Furthermore, hematoxylin and eosin and chitin staining demonstrate that the downregulation of *Lmsinu* led to a prolonged degradation process of the old cuticle during the molting process. Electron microscopy analysis further revealed that knockdown of *Lmsinu* disrupts the formation of septate junctions among epidermal cells, which are a monolayer of polarized epithelial cells, which may hinder the functionality of epidermal cells during the process of molting. In summary, these findings suggest that *Lmsinu* plays a role in nymph molting by regulating the formation of septate junctions among epidermal cells.

## 1. Introduction

Epithelial cells play a pivotal role in the growth and development of insects. They not only undergo differentiation into various tissues, but also significantly contribute to the proper functioning of these tissues [1]. The intestinal epithelial cells function as a protective barrier, effectively blocking the entry of toxins and pathogens into the body [2], and the respiratory epithelial cells can protect the lungs from injury and infection [3]. During epithelial cell formation and proliferation, intercellular junctions are established to facilitate cell–cell and cell–matrix connection. These specialized membrane structures consist of membrane proteins and can be classified into spot adherens junctions, zonula adherens, pleated and smooth septate junctions, gap junctions, and hemiadherens junctions [4,5,6]. The role of cell junctions in the growth and development of insects is pivotal, in which basal spot junctions of epithelial tissues mediate the regulation of Hippo signaling on tissue growth and development [7], and smooth septate junctions are implicated in the regulation of intestinal permeability in arthropods, effectively preventing the leakage of intestinal solutes [8]. The gap junctions are involved in the regulation of metamorphosis and the formation of embryonic gut [9].

The formation of cell junctions involves integral membrane proteins, such as innexin proteins coded by innexin genes in insects for gap junctions [9]. In *Drosophila*, the formation of septate junctions requires the involvement of 33 genes encoding core functional proteins, accessory proteins, and other resident proteins [10]. Mutations or deletions in these genes can disrupt normal tissue functionality in insects. Silencing the expression of the septate junction gene *gliotactin* through RNA interference leads to an augmented intestinal permeability in *Aedes aegypti* [11]. Similarly, knockdown of the *dvssj1* gene (a gene that encodes a membrane protein associated with the smooth septate junction (SSJ) in *Diabrotica virgifera virgifera*) impairs smooth septate junction formation and midgut barrier function in *D. virgifera virgifera* larvae, leading to larval mortality [12]. *Sinuous* is also an important gene involved in the formation of cell junctions. In *Drosophila*, the *sinuous*-encoded claudin plays a crucial role as a key constituent of septate junctions, and *sinuous* mutation would impair the barrier function of septate junctions in salivary glands and cause serious tube size defects [13].

The integument is a vital tissue in insects, serving as both a protective barrier against harmful substances and playing a crucial role in insect growth and development [14,15]. The process of metamorphosis is a crucial indicator of insect growth and development, with molting serving as the primary driving force behind this transformative phenomenon, and the molting process is primarily composed of new epidermis generation and old epidermis degradation [15]. Chitin, lipids, and proteins are important components of insect integument that undergo degradation and re-synthesis during the molting process [16,17,18]. The synthesis and degradation of these epidermal components are primarily regulated by the epidermal cells. During the molting process, epidermal cells are activated by ecdysone and subsequently undergo proliferation. Subsequently, chitin and protein are synthesized within these cells and secreted for depositing new epidermis. After the deposition of new epidermis, chitinase and protease are secreted by the epidermal cells to facilitate the degradation of the old epidermis, thereby accomplishing insect molting [19]. Therefore, the role of epidermal cells in insect molting is crucial, while the epidermal cells are a monolayer of polarized epithelial cells [20]. So, can the presence of sinuous, an essential structural protein of septate junctions, influence molting by modulating epithelial cell functionality?

*L. migratoria* is a prominent agricultural pest [21]. The identification of genes implicated in insect molting presents promising targets for pest control [14,22]. Sinuous is an essential structural protein for cell junctions of epithelial cells, and epithelial cells play an important role in the insect molting process [13,20]. Therefore, it is imperative to investigate whether *sinuous* has an impact on the molting process. In this study, we obtained the *sinuous* gene (*Lmsinu*) from *L. migratoria* transcriptomic data. We investigated the role of the *sinuous* gene in the molting process using RNA interference (RNAi) and evaluated its impact on molting through histological analysis with hematoxylin and eosin (H&E) staining, chitin staining, and transmission electron microscopy. These results will contribute to the advancement of research on the mechanism of locust molting.

## 2. Materials and Methods

### 2.1. Insect Rearing

The locust eggs were obtained from Insect Protein Co., Ltd. (Cangzhou, China). Nymphs were reared in a controlled environment at a temperature of 30 ± 2 °C and relative humidity of 40 ± 5%, following a light/dark cycle of 14 h light and 10 h dark. The nymphs were subsequently nourished with wheat seedlings.

### 2.2. The Sequence Analyses of Lmsinu

The *Lmsinu* gene sequence, encoding the sinuous protein of *L. migratoria*, was obtained from a transcriptome database of *L. migratoria* [23]. By comparing the coding sequence of the *Lmsinu* gene with the locust genome using NCBI BLAST tool, both exons and introns within the *Lmsinu* gene were predicted [21]. The ExPaSy translation tool (http://web.expasy.org/translate/, accessed on 1 October 2020) was utilized to convert the *Lmsinu*-encoding sequence into an amino acid sequence. Conserved protein motifs of the Lmsinu protein sequence were predicted using the SMART tool (http://smart.embl.de/, accessed on 16 June 2023). The prediction of transmembrane domains in the Lmsinu protein was performed using DeepTMHMM version 1.0.24 (https://dtu.biolib.com/DeepTMHMM, accessed on 16 June 2023, Copenhagen, Denmark) [24]. The alignment of sinuous proteins from *L. migratoria* and *D. melanogaster* was conducted utilizing GeneDoc version 2.7.0, a software tool developed by Free Software Foundation (Boston, MA, USA) [25]. Sequence identity analysis of claudin family proteins from different insects was carried out using EMBOSS Water tool (https://www.ebi.ac.uk/jdispatcher/psa/emboss_water, accessed on 16 June 2023, Hinxton, Cambridge, UK) [26]. Accession numbers for claudin family proteins from different insects are presented in Table S1.

### 2.3. The Spatio-Temporal Expression Analysis of Lmsinu

To investigate the expression pattern of *Lmsinu* in different tissues, total RNA was extracted from the foregut, hindgut, midgut, fat body, gastric cecum, hemolymph, integument, and brain of nymphs on the fifth day of the third instar (N3D5). Additionally, total RNA was extracted from the integument of third-instar nymphs to investigate the expression of *Lmsinu* at different stages. The RNAiso Plus kit (TaKaRa, Tokyo, Japan) was used for total RNA extraction following the manufacturer’s protocol. The NanoDrop 2000 spectrophotometer (Thermo Fisher Scientific Inc., Waltham, MA, USA) was utilized for quantifying the RNA concentration. The MonScript™ RTIII All-in-One Mix with dsDNase kit (Monad Biotech Co., Ltd., Wuhan, China) was employed to synthesize the first-strand cDNA. The reverse transcription system comprises a total volume of 20 μL with sequential addition of the following reagents: 1 μg of RNA, 1 μL of MonScript™ 5× RTIII All-in-One Mix, and 1 μL of MonScript™ dsDNase. Finally, RNase-free water was added to adjust the final volume to 20 μL. The reverse transcription protocol was as follows: incubation at 37 °C for 2 min, followed by incubation at 55 °C for 15 min, and finally, incubation at 85 °C for 5 min.

The BlasTaqTM 2X qPCR MasterMix kit (ABM Inc., Nanjing, China) was employed to conduct the quantitative polymerase chain reaction (qPCR) on a LightCycler^®^ 480 Real-Time PCR System (Roche Diagnostics GmbH, Mannheim, Germany). The reaction system comprised 10 μL of BlasTaqTM 2X qPCR MasterMix, 3 μL of cDNA, 1 μL each of positive and negative primers (10 μM), and 6 μL of nuclease-free H_2_O. The qPCR protocol began with a 5 min pre-denaturation step at 95 °C, followed by 40 cycles of denaturation at 95 °C for 15 s and annealing/extension at 60 °C for 30 s. Finally, the melting curve for each pair of primers was analyzed. The 2^−ΔΔCt^ method was utilized to determine the transcriptional expression of *Lmsinu* [27], and the housekeeping gene *LmEF-1α* was employed as an internal reference to standardize the expression level of *Lmsinu* [28]. The experimental design consisted of 3 independent biological replicates for each sample, and 3 nymphs were involved in each replicate. The primers employed in the experiment can be found in Table S1.

### 2.4. The Functional Analysis of Lmsinu through RNA Interference (RNAi)

To investigate the biological function of *Lmsinu*, the PCR products of *GFP* (green fluorescent proteins) and *Lmsinu* genes were employed as templates for the synthesis of double-stranded RNA (dsRNA). The T7 RiboMAX™ Express RNAi System (Promega, Inc., Madison, WI, USA) was employed to synthesize dsRNAs targeting *GFP* (ds*GFP*) and *Lmsinu* (ds*Lmsinu*) following the manufacturer’s protocol. Next, 6 μg and 10 μg of dsRNAs were injected into the nymphs through their abdomen on the first day of the third and fourth instar, respectively. After a 48 and 96 h period following dsRNA injection, we isolated the integument from the treated nymphs and extracted total RNA, which was then used to evaluate the silencing efficiency by RT-qPCR. A total of three independent biological replicates were prepared, each replicate consisting of three nymphs. Additionally, after dsRNA injection, we reared the nymphs under normal conditions for subsequent phenotypic investigation while using a group injected with ds*GFP* as a control. The primers employed in the experiment can be found in Table S1. The dsRNA sequence of *Lmsinu* is shown in File S3.

### 2.5. Microsection and Hematoxylin and Eosin (H&E) Staining of Integument

To further investigate the impact of *Lmsinu* RNAi on the process of molting, the integument of the third-instar larvae was stained with H&E as described previously [29]. In brief, on the first day of the third instar, 6 μg of ds*GFP* and ds*Lmsinu* were injected into the nymphs. On the fifth day of the third instar (N3D5), we dissected the integuments of the second abdominal segment from the nymphs injected with ds*GFP* and ds*Lmsinu*. Subsequently, the dissected integuments were fixed and utilized for the preparation of paraffin sections (5 μm in thickness), followed by staining with hematoxylin and eosin. The transections of the stained integument were examined under an Olympus BX51 microscope (Olympus, Tokyo, Japan) and captured with an Olympus digital camera. According to the H&E staining images, we utilized ImageJ software (version 1.53t, National Institutes of Health, Maryland, USA) for quantifying the thickness of the old cuticle of the second abdominal segment from ds*GFP*- or ds*Lmsinu*-injected nymphs on the fifth day of the third instar. The experimental design included 10 independent biological replicates for each sample.

### 2.6. Microsection and Chitin Staining of Integument

To investigate the effect of the silencing of *Lmsinu* on the chitin of the integument, microsections and chitin staining of the integuments were performed following previously described methods [30]. In brief, on the fifth day of the third instar, the integuments of the second abdominal segment from nymphs injected with ds*GFP* or ds*Lmsinu* were prepared as 5 μm paraffin sections. Subsequently, chitin was stained with Fluorescent Brightener 28 (Sigma Inc., St. Louis, MO, USA) at a concentration of 1 mg/mL, while the nucleus was counterstained using SYTOX™ Green nucleic acid stain (Thermo Fisher Scientific, Waltham, MA, USA) at a concentration of 25 μg/mL [31]. Images of the transection of the stained integument were captured using an LSM 880 confocal laser scanning microscope (Zeiss, Inc., Oberkochen, Germany).

### 2.7. Transmission Electron Microscopy of Integument

To further investigate the alterations in the septate junctions among epidermal cells subsequent to *Lmsinu* silencing, the ultrastructural analysis of the integuments was conducted using transmission electron microscopy (TEM) following previously described methods [32]. Firstly, on the fifth day of the third instar, the integuments of the second abdominal segment from the nymphs injected with ds*GFP* or ds*Lmsinu* were dissected, and the dissected integuments were then fixed in a 0.2 M phosphate-buffered solution (pH7.2) containing 3% glutaraldehyde at 4 °C for 48 h. Subsequently, the preliminarily fixed integuments were rinsed thrice with phosphate buffer solution, followed by subsequent fixation in 1% osmium tetroxide at 4 °C for a duration of 3 h. Then, the fixed integuments were rinsed twice, each time for a duration of 10 min. The samples were subsequently dehydrated using varying concentrations of acetone (10%–100%) and embedded in Epon 812. The samples were subsequently sectioned into ultrathin slices, which were then carefully collected onto copper grids. A JEM-1200EX transmission electron microscope (TEM, JEOL, Tokyo, Japan) was utilized for capturing the images of the transection of the integument.

### 2.8. Data Analysis

The SPSS software (version 19.0; SPSS Inc., Chicago, IL, USA) was utilized for the statistical analyses. Tukey’s HSD multiple comparison test was employed to compare the expression level of *Lmsinu* in different developmental stages and tissues, while the independent sample *t*-test was utilized for analyzing other data.

## 3. Results

### 3.1. Bioinformatic Analysis of Lmsinu

**Figure 1:**
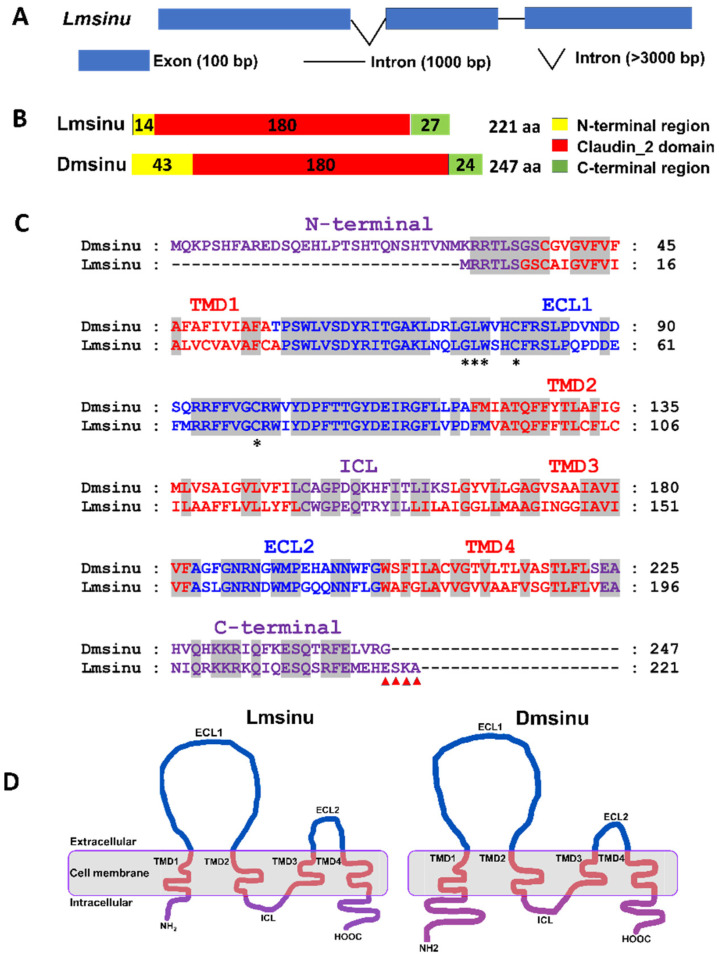
Sequence analysis of *Lmsinu* in *L. migratoria*. (**A**) Genomic architecture of *Lmsinu*. The exons are represented by blue boxes, while the introns are depicted as black lines in the genomic structure of *Lmsinu*. (**B**) Conserved domains of Lmsinu with Dmsinu. The numbers within the frame represent the count of amino acids within each respective domain, with distinct colors are assigned to different domains. The sequences were obtained from *L. migratoria* (Lm) and *D. melanogaster* (Dm), respectively. “aa” denotes amino acid. (**C**) Multiple sequence alignments of Lmsinu and Dmsinu protein sequences. TMD: transmembrane domain, ECL: extracellular loop, ICL: intracellular loop. The asterisked amino acids represent the conserved residues within the claudin family proteins, and the amino acids labeled with red triangles represent a PDZ-binding motif. The amino acids located extracellularly, within the cell membrane, and intracellularly are, respectively, depicted in blue, red, and purple hues. The sequences displayed on the gray background exhibit complete conservation. (**D**) Diagram depicting the binding of Lmsinu and Dmsinu proteins to the cell membrane (depicted in grey). The different domains of Lmsinu and Dmsinu proteins located extracellularly, within the cell membrane, and intracellularly are, respectively, depicted in blue, red, and purple hues. The explanations for ECL, TMD, and ICL remain consistent with those depicted in (**C**).

The *sinuous* gene was obtained based on the transcriptome of *L. migratoria* and was named as *Lmsinu*. It consists of three exons and two introns (Figure 1A). The coding region of *Lmsinu* spans a length of 666 bp and encodes a total of 221 amino acids (Files S1 and S2). Similar to the *Drosophila* sinuous (Dmsinu) protein, the Lmsinu protein sequence also exhibits a conserved claudin2 domain (Figure 1B). Moreover, the Lmsinu protein comprises four transmembrane domains and two extracellular domains, which is consistent with the Dmsinu protein (Figure 1C,D). Additionally, when comparing the amino acid sequences of claudin proteins from *L. migratoria* and other insects, it was found that the amino acid sequence identity between the Lmsinu protein and sinuous proteins from other insects was as high as 52.3% to 76.5% (Table S2).

### 3.2. Analysis of Spatiotemporal Expression Pattern of Lmsinu

**Figure 2:**
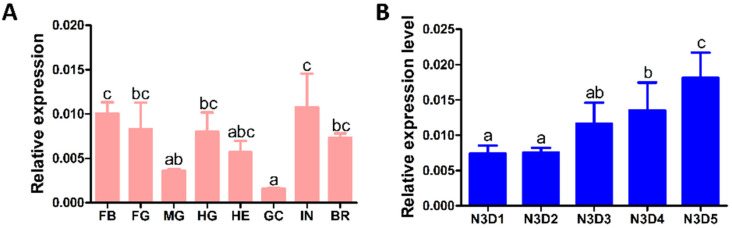
The expression patterns of *Lmsinu* in various developmental stages and tissues. (**A**) The expression pattern of *Lmsinu* in various tissues of fifth-day nymphs at the third instar. FB: fat body; FG: foregut; MG: midgut; HG: hindgut; HE: hemolymph; IN: integument; BR: brain. (**B**) The expression pattern of *Lmsinu* in nymphs during third instar stage. N3D1-N3D5: from day 1 to day 5 of the third instar. The data were subjected to analysis using Tukey’s HSD multiple comparison test. The distinct letters above each column indicate statistically significant differences among the various samples (*p* < 0.01).

First, on the fifth day of the third instar, the expression of *Lmsinu* in various tissues of the nymphs was examined. The results revealed that *Lmsinu* exhibited the highest level of expression in the integument and fat body, while its expression level was found to be the second highest in the foregut, hindgut, and brain (Figure 2A). Moreover, alterations in the expression level of *Lmsinu* were investigated in the integument at the third instar. The expression level of *Lmsinu* exhibited a gradual increase during the third instar period, reaching its peak on the final day of this developmental stage (Figure 2B).

### 3.3. The Impact of Lmsinu Silencing on the Molting Process of Nymphs

**Figure 3:**
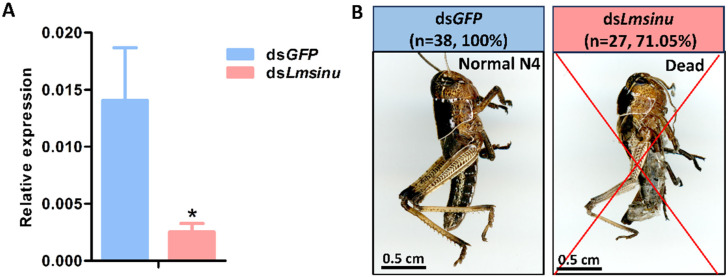
The impact of the silencing of *Lmsinu* on the molting process of third-instar nymphs of *L. migratoria*. (**A**) Expression analysis of *Lmsinu* after the injection of ds*GFP* and ds*Lmsinu* for 48 h. The data were subjected to analysis using the independent-samples *t*-test, with asterisks denoting statistically significant differences between the groups injected with ds*GFP* and ds*Lmsinu* (*p* < 0.05). (**B**) Phenotypic observations following the silencing of *Lmsinu*. The aforementioned percentage represents the proportion of individuals that undergo mortality during the molting process from the third instar to the fourth instar. N4: fourth-instar nymph.

To explore the effect of the silencing of *Lmsinu* on the molting process of *L. migratoria*, double-stranded RNA (ds*Lmsinu*) was synthesized utilizing a 389 bp fragment of *Lmsinu* as a template; as a control, double-stranded RNA (ds*GFP*) was synthesized utilizing the green fluorescent protein gene as a template. We injected ds*GFP* and ds*Lmsinu* on the first day of the third instar; after 48 h of dsRNA injection, the expression of *Lmsinu* exhibited a significant reduction by 82.01% compared to the control group (Figure 3A). The nymphs injected with ds*GFP* successfully molted to the fourth instar, while 71.05% of the nymphs injected with ds*Lmsinu* died during the molting process (Figure 3B). Moreover, ds*GFP* and ds*Lmsinu* were also injected into nymphs on the first day of the fourth instar, and the expression level of *Lmsinu* was significantly decreased by 96.40% compared to the control group after 48 h (Figure S1A), while 66.67% of the nymphs perished on the fourth instar due to unsuccessful molting (Figure S1B).

### 3.4. The Silencing Effects of Lmsinu on Nymphal Molting

**Figure 4:**
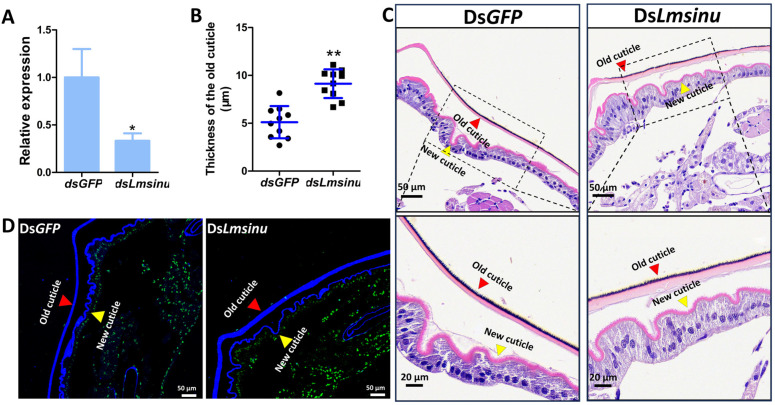
The impact of the silencing of *Lmsinu* on the microstructure of the integument. (**A**) Expression analysis of *Lmsinu* after the injection of ds*GFP* and ds*Lmsinu* for 96 h. The expression level of *Lmsinu* in the control group was normalized to 1. The data were subjected to analysis using the independent-samples *t*-test, with asterisks denoting statistically significant differences between the groups injected with ds*GFP* and ds*Lmsinu* (*p* < 0.05). (**B**) Thickness analysis of the old cuticle of the second abdominal segment from ds*GFP*- or ds*Lmsinu*-injected nymphs on the fifth day of the third instar. The data are presented as the means ± standard error (SE) of ten independent biological replications. Statistical significance was assessed using the independent-samples *t*-test. ** *p* < 0.01. (**C**) Microstructural analysis of the integuments of the second abdominal segment from ds*GFP*- or ds*Lmsinu*-injected nymphs on the fifth day of the third instar using H&E staining. The red and yellow triangles, respectively, represent the old cuticle and the new cuticle. (**D**) Microstructural analysis of the integuments of the second abdominal segment from ds*GFP*- or ds*Lmsinu*-injected nymphs on the fifth day of the third instar using chitin staining. The nucleus was labeled with SYTOX™ Green nucleic acid stain (Thermo Fisher Scientific, Waltham, MA, USA), exhibiting a green fluorescence signal, while chitin was stained using Fluorescent Brightener 28 (Sigma Inc., St. Louis, MO, USA), resulting in a blue coloration. (**C**,**D**) were both taken from the transection of the integument.

To investigate the impact of ds*Lmsinu* injection on microstructural alterations during locust molting, on the fifth day of the third instar, we stained the integuments of nymphs injected with ds*GFP* and ds*Lmsinu* using H&E and chitin staining. The results showed that, after 96 h of dsRNA injection, the expression of *Lmsinu* exhibited a significant reduction by 66.65% compared to the control group (Figure 4A). The old cuticle of the nymphs injected with ds*Lmsinu* was thicker than that of the nymphs injected with ds*GFP* on the fifth day of the third instar (Figure 4C,D). The thickness of the old cuticle of the second abdominal segment from nymphs injected with ds*Lmsinu* was 1.79-times greater compared to that of nymphs injected with ds*GFP* (Figure 4B).

### 3.5. Effects of the Silencing Lmsinu on Cell Junctions in Epidermal Cells

**Figure 5:**
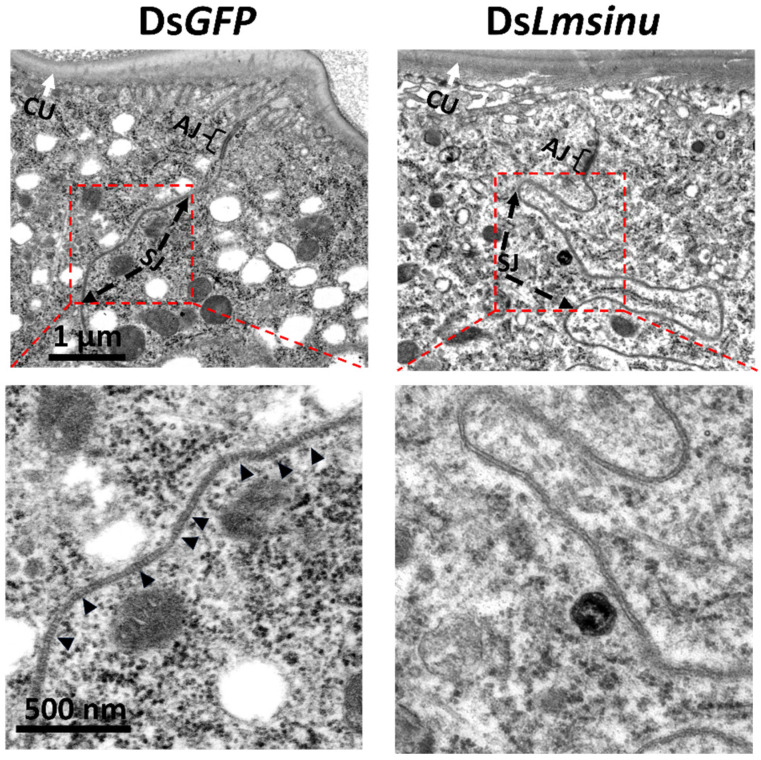
TEM analysis of the integuments of the second abdominal segment from nymphs injected with ds*GFP* or ds*Lmsinu*. The region enclosed by the large brackets represents the adherens junction (AJ); the region indicated by the black arrow corresponds to the septate junction (SJ); the black triangles represent the septa within the septate junctions; the white arrow points towards the cuticle (CU). The images were taken from the transection of the integument.

*Sinuous* has been identified as a crucial constituent of septate junctions among insect epithelial cells [33], so we employed transmission electron microscopy to examine the cell junctions among epidermal cells of ds*GFP*- and ds*Lmsinu*-injected nymphs on the fifth day of the third instar. The results showed that the silencing of *Lmsinu* exerted a significant influence on the establishment of septate junctions. Normal septa were observed within the septate junctions of epidermal cells from ds*GFP*-injected nymphs, but there were no obvious septa in the septate junctions of epidermal cells from ds*Lmsinu*-injected nymphs (Figure 5).

## 4. Discussion

Epidermal cells play a pivotal role in the molting process of insects; during the process of insects molting, epidermal cells can secrete chitinase and protease to degrade the old cuticle, and can also secrete proteins and phenols to synthesize the new cuticle [19]. Epidermal cells are a monolayer of polarized epithelial cells; the epithelial cells are connected by a specialized structure known as cell junctions; the crucial role of cell junctions in cellular functioning has been extensively documented [4,6,20]. Sinuous is a crucial structural protein of the cell junctions [33]. Could it potentially impact insect molting by modulating the cell junctions of epidermal cells?

In the present study, we identified the sinuous gene from *L. migratoria*, and its protein sequence contains the conserved claudin2 domain, which proves that the Lmsinu protein belongs to the claudin family. Most claudin proteins are structural components of cell junctions and consist of four transmembrane domains, one intracellular loop (LCL), and two extracellular loops (ECL) [34]. Similarly, Lmsinu protein is also a tetraspan transmembrane protein, which is consistent with the structure of claudin proteins. The claudin family proteins exhibit conserved amino acid motif W-GLW-C-C in the extracellular loop and possess PDZ binding sites at their C-terminus [35]. In the Lmsinu protein sequence, we have also identified the characteristic motif W-GLW-C-C in ECL, along with a PDZ binding site ESKA at its C-terminus (Figure 1C). These findings provide further evidence supporting the classification of Lmsinu as a member of the claudin family. The claudin family proteins documented in insects are predominantly identified in the Diptera order, encompassing fruit flies and mosquitoes. For example, the claudin proteins, sinuous, megatrachea, and kune-kune have been successfully cloned from both *D. melanogaster* and *A. aegypti* [33,36,37,38]. Sequence comparison in our study revealed remarkably high identity between the Lmsinu and sinuous proteins of *D. melanogaster* (62.6%), as well as *A. aegypti* (61.4%) (Table S1), which far exceeded the resemblance observed between Lmsinu and other claudin family proteins found in insects. Overall, these findings substantiate the validity of identifying the sinuous gene in *L. migratoria*.

Sinuous is currently reported to be an important component of septate junctions in *D. melanogaster* [33]. In invertebrates, septate junctions are predominantly observed in ectodermal-derived epithelia, such as the integument, foregut, and hindgut [10,39]. In the present report, we initially investigated the expression of *Lmsinu* in various tissues and found relatively higher levels of *Lmsinu* expression in the epidermis, foregut, and hindgut compared to other tissues. The expression site of *Lmsinu* corresponds to the anatomical location of the septate junctions in invertebrates, which provides some evidence that *Lmsinu* is an important component of septate junctions. Furthermore, the expression of *Lmsinu* in the integument of nymphs during third instar was analyzed. During the third instar, *Lmsinu* exhibited relatively low expression levels during the early and middle stages, gradually increasing to its peak towards the end of this developmental stage. At the end of each instar, insects initiate preparatory processes for molting by upregulating numerous pivotal genes associated with molting. The chitinase (cht) gene plays a pivotal role in the process of insect molting; the expression level of the cht-5 gene in *Hyphantria cunea* and *Sogatella furcifera* is significantly upregulated prior to larval molting [40,41]. In addition, nuclear transcription factors involved in regulating key molting genes show heightened expression during the pre-molting phase. For example, in *Blattella germanica*, *Leptinotarsa decemlineata*, and *Nilaparvata lugens,* hormone receptor 4 (HR4) and FTZ-F1 serve as pivotal regulators of the 20E signaling pathway, exhibiting a substantial upregulation in the expression level during the pre-molting stage [42,43,44]. Therefore, the high expression of *Lmsinu* on the eve of molting implies its potential involvement in the molting process of *L. migratoria*.

To elucidate the physiological role of *Lmsinu* in the growth and development of *L. migratoria*, RNAi was employed to investigate its functional significance. The silencing of *Lmsinu* was observed to induce molting difficulties, resulting in increased nymph mortality during the molting process. The function of the sinuous gene has thus far only been documented in *D. melanogaster*, where it plays a crucial role in septate junction formation, and its mutation leads to severe defects in tube size regulation [13]. While no reports have indicated a correlation between the *sinuous* gene and insect molting, numerous studies have demonstrated that the absence of the septate junction-related proteins can significantly impact insect molting. For example, in *Oncopeltus fasciatus*, silencing the Na, K-ATPase gene impacted septate junction formation and ultimately influenced the molting process [45]. Snakeskin is a membrane protein related to septate junctions; in *Henosepilachna vigintioctopunctata*, knockdown of the *snakeskin* gene using RNA interference during the third-instar larval stage halted larval development with most resulting larvae unable to molt until they die [46]. Rab11 is an essential small GTPase for the formation of septate junctions; silencing *LmRab11A* would arrest the molting process in *L. migratoria* [47,48]. These observations suggest that septate junction-related proteins play a pivotal role in insect molting, aligning with the evidence that silencing the *Lmsinu* gene leads to impaired molting in *L. migratoria*.

Moreover, we conducted a comprehensive analysis of the structural alterations in the integument following the silencing of *Lmsinu*. Our findings revealed that the old cuticle of nymphs injected with ds*Lmsinu* exhibited a significantly increased thickness compared to that of nymphs injected with ds*GFP* prior to molting, which implies that silencing *Lmsinu* delayed the molting process of locusts. The protein sinuous plays a pivotal role in the formation of septate junctions among epithelial cells [33]. Epidermal cells are a monolayer of polarized epithelial cells [20], which not only secrete chitinase and protease for degrading the old cuticle, but also synthesize necessary substances for new cuticle formation during molting [19]. Therefore, silencing *Lmsinu* may potentially affect epidermal cell functionality, thereby impacting the molting process. Additionally, transmission electron microscopy analysis demonstrated that silencing *Lmsinu* resulted in an absence of septa within septate junctions of epidermal cells, consistent with the phenotype observed after silencing sinuous in *D. melanogaster*, where it also impedes septa formation within septate junctions [33]. Genes involved in the formation of septate junctions are crucial for maintaining the apical–basal polarity of epithelial cells [49]. For example, *yurt* is a crucial gene encoding a septate junction protein, and its mutation can disrupt the apical–basal polarity in tracheal epithelium during mid-embryogenesis [49,50]. Insect epidermal cells are polarized monolayer cells, which are apically attached to the cuticle, while they are basally attached to a poorly developed basement membrane [51]. The apical–basal polarity of epidermal cells is linked to insect molting; in particular, the apical surface of epidermal cells plays a crucial role in both the secretion of insect molting-related substances and the deposition of new epidermis [52]. *Sinuous* mutation has been reported to induce alterations in the apical layer of epithelial cells. In *D. melanogaster*, *sinuous* mutation also caused an abnormal apical layer of tracheal epithelial cells [33]. Therefore, *Lmsinu* may modulate the formation of septate junctions among epidermal cells, thereby exerting an influence on the polarity and functionality of epidermal cells, consequently impacting insect molting.

## 5. Conclusions

In this study, the *sinuous* gene was identified from *L. migratoria* and found to be a member of the claudin family proteins. Temporal and spatial expression analysis revealed that *Lmsinu* exhibited relatively high expression levels in the cuticle, fat body, foregut, midgut, and brain. Additionally, its expression significantly increased just prior to molting. Furthermore, RNAi technology was employed to investigate the biological function of *Lmsinu* in *L. migratoria*, demonstrating that silencing *Lmsinu* resulted in larval mortality during molting. Histological examination (HE) and chitin staining indicated that the silencing of *Lmsinu* resulted in delayed degradation of the old cuticle during molting. Moreover, electron microscopy experiments revealed that the silencing of *Lmsinu* disrupted the formation of septate junctions among epidermal cells. In summary, these findings suggest that *Lmsinu* is involved in nymph molting by regulating the formation of septate junctions among epidermal cells during the molting process in *L. migratoria*.

## Data Availability

The original contributions presented in the study are included in the article/Supplementary Materials, further inquiries can be directed to the first and corresponding author.

## Supplementary Materials

The following Supporting Information can be downloaded at: https://www.mdpi.com/article/10.3390/genes15070850/s1, Figure S1: The impact of the silencing of *Lmsinu* on the molting process of fourth-instar nymphs in *L. migratoria*. (A) The expression analysis of *Lmsinu* after injection of ds*GFP* and ds*Lmsinu* for 48 h. The data underwent analysis using the independent-samples *t*-test, with asterisks denoting statistically significant differences between the groups injected with ds*GFP* and ds*Lmsinu* (*p* < 0.05). (B) Phenotypic observations following the silencing of *Lmsinu*. The aforementioned percentage represents the proportion of individuals that undergo mortality during the molting process from the third instar to the fifth instar stage. N5: fifth-instar nymph; Table S1: Primers used in the experiments; Table S2: Identity and similarity analysis of insect claudin family proteins; File S1: The CDS sequence of *Lmsinu*.fa; File S2: The protein sequence of *Lmsinu*.fa; File S3: The dsRNA sequence of *Lmsinu*.fa.
