# Supplementary material for: Sinuous Is a Claudin Required for Locust Molt in Locusta migratoria
Source: Genes (Basel). 2024 Jun 27;15(7):850. doi: 10.3390/genes15070850 (PMC11275452; doi:10.3390/genes15070850)
Supplement: Supplementary file 1 [file genes-15-00850-s001.zip › genes-3065871-supplementary.pdf]

Table S1. Primers used in experiments

Primer name	Sequence (5'-3')	Application
Lmsinu-RNAiF	TAATACGACTCACTATAGGGCGTTGGATGCAGGTGGATAT	RNAi
Lmsinu-RNAiR	TAATACGACTCACTATAGGGTCGCCTCTACCAGAAATAAG	RNAi
LmGFP-RNAiF	TAATACGACTCACTATAGGGCAGTTCTTGTTGAATTAGATG	RNAi
LmGFP-RNAiR	TAATACGACTCACTATAGGGTTTGGTTTGTCTCCCATGATG	RNAi
Lmsinu-qF	TTTGTGCACCAAGTTGGCTT	qPCR
Lmsinu-qR	CGGCAAGGACCGAAAACAATG	qPCR
LmEF-1α-qF	AACATCGTCGTCATTGGTCA	qPCR
LmEF-1α-qR	GTTCAGCCTTCAGCTTGTCC	qPCR

Table S2. Identity and similarity analysis of insect Claudin family proteins

Gene	Accession numbers	Identity
Dmsinu	NP_647971.3	62.6%
Dmkune	NP_610179.2	39.9%
Dmmega	NP_569919.2	29.2%
DmCG45049	NP_001097876.1	25.0%
DmCG6398	NP_001285391.1	19.8%
Agsinu	DAA64999.1	61.4%
Agmega	DAA64998.1	25.5%
Pxsinu	AEN04482.1	66.7%
Dpsinu	OWR49408.1	61.8%
Adsinu	ETN66490.1	63.6%
Cqsinu	EDS35653.1	62.7%
Ctsinu	JAV29021.1	62.3%
Obsinu	KOB73465.1	52.3%
Tnsinu	QCP68938.1	76.5%
Pxusinu	BAM19606.1	65.9%

Dm: *Drosophila melanogaster*, Ag: *Aedes aegypti*, Px: *Plutella xylostella*, Dp: *Danaus plexippus*, Ad: *Anopheles darlingi*, Cq: *Culex quinquefasciatus*, Ct: *Culex tarsalis*, sinu: Ob: *Operophtera brumata*, Tn: *Trichoplusia ni*, Pxu: *Papilio xuthus*, Sinuous, kune: Kune-kune, mega: Megatrachea.

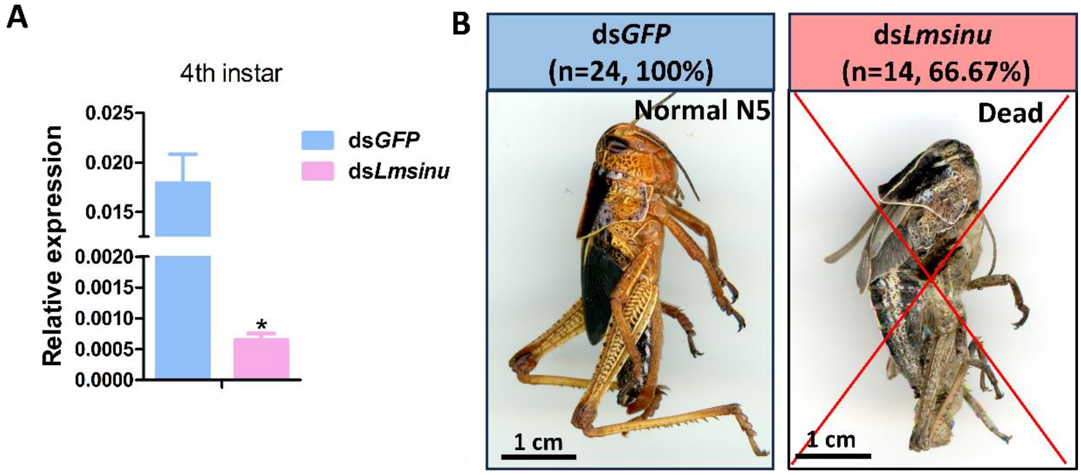

Figure S1. The impact of the silencing of *Lmsinu* on the molting process of fourth instar nymphs in *L. migratoria*. (A) The expression analysis of *Lmsinu* after injection of ds*GFP* and ds*Lmsinu* for 48 hours. The data underwent analysis using the independent-samples T-test, with asterisks denoting statistically significant differences between the groups injected with ds*GFP* and ds*Lmsinu* ($p<0.05$). (B) Phenotypic observations following the silencing of *Lmsinu*. The aforementioned percentage represents the proportion of individuals that undergo mortality during the molting process from the third instar to the fifth instar stage. N5: fifth instar nymph.
